# The physical activity levels among people living with human immunodeficiency virus/acquired immunodeficiency syndrome receiving high active antiretroviral therapy in Rwanda

**DOI:** 10.1080/17290376.2014.886081

**Published:** 2014-02-13

**Authors:** J.M. Frantz, A. Murenzi

**Affiliations:** a PhD, is a professor in the Department of Physiotherapy at the University of the Western Cape, Private Bag x17, Bellville 7530, South Africa; b MSc, is a physiotherapist and masters graduate in the Department of Physiotherapy at the University of the Western Cape, Private Bag x17, Bellville 7530, South Africa

**Keywords:** HIV, AIDS, physical inactivity, Rwanda, HAART, VIH, SIDA, l'inactivité physique, le Rwanda, la multithérapie

## Abstract

The accessibility of high active antiretroviral therapy (HAART) for local human immunodeficiency virus (HIV) patients is improving in Rwanda. It is well known that this therapy is associated with serious adverse effects, such as metabolic and morphologic changes. One of the recommended preventive modalities for these complications is participation in physical activity. The current study aims to determine the anthropometric profile and physical activity levels among people living with HIV and receiving HAART in Kigali, Rwanda. The study was a cross-sectional, descriptive quantitative survey. The participant's levels of physical activity participation and their association with anthropometric profiles were measured, using a structured self-administered questionnaire for 407 clients passing through the clinics. Of the participants, approximately 70% were inactive and in addition, 40% were obese and 43% overweight. Obesity was found to be strongly associated with inactivity. Lack of motivation, and time as well as fear of worsening the disease were found to be barriers to participation in physical activity.

## Résumé

L'accessibilité de la multithérapie antirétrovirale pour les patients locaux du VIH s'améliore au Rwanda. Il est bien connu que cette thérapie est associée à des effets indésirables graves, tels que métaboliques et morphologiques changements. Une des modalités de prévention recommandées pour ces complications est de l'activité physique. La présente étude a comme but de déterminer le profil anthropométrique et niveaux d'activité physique chez les personnes vivant avec le VIH et recevant une multithérapie à Kigali, Rwanda. L'étude était une enquête quantitative transversale, descriptive. Les niveaux de participation à l'activité physique et leur association avec des profils anthropométriques des participants ont été mesurés à l'aide d'un questionnaire auto – administré structuré pour 407 clients en passant par les cliniques. Parmi les participants, environ 70% des participants étaient inactifs et en plus, 40% et 43% étaient obèses et en surpoids respectivement. L'obésité a été trouvé à être fortement lié à l'inactivité. Manque de motivation, le temps et la peur de l'aggravation de la maladie ont été parmi les obstacles à la participation en surbrillance de l'activité physique.

## Introduction

In developed countries, people living with human immunodeficiency virus (HIV)/acquired immunodeficiency syndrome (AIDS) are living longer, thanks to the accessibility of treatment (UNAIDS 2009). Developing countries, on the other hand, are heavily affected by HIV infection, with a prevalence rate of 90% of all people living with HIV in the world (Global Health Council 2010). Currently, 70% of people (22.4 million) living with HIV worldwide are found in Sub-Saharan African countries. The worldwide accessibility of high active antiretroviral therapy (HAART) has increased gradually worldwide since the end of 2008. According to WHO (2010), people on HAART accounted for approximately 4 million people worldwide, and 2.9 million of them were in Sub-Saharan Africa. In Rwanda, the proportion of people infected by HIV during 2005 was 3% in the general population aged 15–49 years (DHS Rwanda 2005). Causes suggested included the genocide of 1994, when many women were raped, sexually tortured and psychologically traumatised (CNLS Rwanda 2007).

Rwanda is one of the countries sponsored by the US president's emergency plan for AIDS relief. This donation has assisted in increasing accessibility to HAART by Rwandan HIV patients. In Rwanda, more than 70% of people in need of antiretroviral therapies (HAART) are being treated (UNAIDS 2009). The use of HAART has been identified as having several advantages, particularly greater longevity of people living with HIV/AIDS (Centres for Disease Control and Prevention 2003). Research has, however, shown that the use of HAART is significantly associated with harmful changes, particularly morphologic and metabolic changes (Vergara-Rogriguez, Vibhakar & Watts 2009). Furthermore, the presence of obesity, dietary imbalances and sedentary lifestyles, aggravates these metabolic disturbances (Shah, Tierney & Adams-Huet 2005). Obesity has been identified as a key risk factor for coronary heart diseases and type 2 diabetes mellitus when correlated with physical inactivity among people living with HIV/AIDS under HAART (Amorosa, Synnestvedt, Gross, Friedman, Mac-Gregor & Gudonis 2005).

In the last decade, participation in physical activity has been identified as one way to prevent and manage the adverse effects of HAART (Aberg 2003; Amorosa et al. 2005), which can include cardiovascular disease, lipodystrophy and glucose or lipid metabolism complications (Malita, Karelis, Toma & Rabasa-Lhoret 2005; Terry, Sprinz, Stein, Medeiros, Oliviera & Ribeiro 2006). Several other studies have reported that the benefits of physical activities/exercises in people living with HIV/AIDS while being treated with HAART include the improvement of their quality of life and well-being (Florindo, Latorre, Jaime & Segurado 2007; Mutimura, Stewart, Crowther, Yarasheski & Cade 2008), their strength (Roubenoff & Wilson 2001), and their increasing functional work capacity (Hand, Philips, Dudgeon, Lyerly, Dustine & Burgess 2007). The other benefits indicated are psychological effects (Roubenoff 2000) and self-efficacy (Fillipas, Oldmeadow, Bailey & Cherry 2007). In addition, physical fitness and the ability to perform activities of daily living or an improvement of the body's ability to fight infection as well as a slower progression of HIV to AIDS were found to be some of the benefits of physical activity (Bopp, Fillipas, Fulk & Hand 2003; Robinsoni, Quinn & Rimmer 2007).

There is limited information available on the levels and effects of physical activity on HIV/AIDS patients in Sub-Saharan Africa. One study conducted by Mutimura et al. (2008) has confirmed the positive effects of physical activity among HIV-infected people treated with HAART, especially on metabolic and cardiovascular-related risks. However, the literature indicates that despite the clear benefits of physical activity for persons living with HIV/AIDS, studies on engagement in adequate physical activity are still limited (Clingerman 2004; Farah, Barbara, Nelson & Jorge 2004).

According to the World Bank Report, physical activity needs to be promoted in all domains, as the levels of inactivity are high in both developed and developing countries. With the increase of HIV/AIDS, more people, particularly HIV patients, are increasingly less involved in physical activity. There is, therefore, a need to encourage patients with HIV to participate in physical activities because they are often not motivated to exercise (Buchholz & Purath 2007). Several studies around levels of physical activity have been conducted on different groups In Rwanda, including university employees (Banyangiriki 2009), adults living with diabetes mellitus (Kabanda & Phillips 2011), university students (Tumusiime & Frantz 2006) and women working in high-income institutions (Kagwiza, Philips & Struthers 2005). All these studies have indicated that the majority of participants were physically inactive, highlighting the risk of developing non-communicable diseases such as cardiovascular problems and diabetes mellitus. The challenge for researchers is to determine whether there is a similar situation among people living with HIV/AIDS while being treated with HAART.

Currently in Rwanda, the accessibility of HAART for local HIV patients is increasing. It is well known that this therapy is associated with serious adverse effects, such as metabolic and morphologic changes. These effects can be prevented and/or treated by engaging in regular physical activity. However, according to Rwandan literature, levels of physical activity in the general population are low. The aim of the present study was thus to determine the anthropometric profile and physical activity levels of people in Rwanda living with HIV/AIDS while being treated with HAART. This information may be a useful tool in the implementation of promotion strategies aimed at the prevention and appropriate management of people living with HIV/AIDS treated with HAART and those at risk of developing cardiovascular and diabetic diseases.

## Methodology

### Research design

A quantitative cross-sectional descriptive study design was used in the present study.

### Population and sampling

The study population included all adults, aged above 18 years, living with HIV/AIDS, who have been on HAART regimens for at least 12 months, and who are attending three health centres in Rwanda. During the data collection period, it was estimated that approximately 647 patients would pass through the three health centres during the data collection period. According to Yamane's formula (Israel 1992), where (*n*) stands for sample; (*N*) for study population, and (*e*) is equal to 0.05, a representative sample size of the HIV patients would be 407 participants. Based on the information of patients passing through the various clinics, a proportionate sample was determined and thus, 92 participants were targeted from Centre A, 137 participants from Centre B, and 178 from Centre C. On the day that the researcher was present, a convenient sample of people attending the clinic was approached to participate in the study until the target number was reached. All these participants voluntarily agreed to take part in the study.

### Instrumentation

Data were collected by means of a structured self-administered questionnaire. The questionnaire consisted of three sections which were composed of closed-ended questions. Section A focused on the socio-demographic data of the participants and consisted of eight items. These items included age, gender, marital status, level of education and occupation. In this section, the medical profiles, including duration on HAART, opportunistic diseases and CD4+ cell counts were also recorded, to describe the medical status of patients. Section B consisted of anthropometric measurements. These measurements included height, weight, and waist and hip circumferences. Weight and height were measured using a calibrated digital scale and tape measure nearest to 0.1 kg and 0.1 cm respectively. Participants stood, wearing light clothes and no shoes. Waist and hip circumferences were measured using a non-stretch cloth tape-measure. To ensure reliability, the participants’ anthropometric profiles were taken twice by the same research assistant, and if these differed by 2 cm, a third measurement was taken and the average was used. These measurements enabled the researcher to get the anthropometric profiles such as Body Mass Index (BMI), waist circumference, and waist-hip ratio (WHR). Section C of the questionnaire addressed the types and levels of physical activity participation, using the adapted Sub-Saharan African Activity Questionnaire (SSAAQ) designed by Sobngwi, Mbanya, Unwin, Aspray and Albert (2001). The SSAAQ is the most appropriate to use in Sub-Saharan populations (Sobngwi *et al.* 2001). The questionnaire was modified to suit persons with HIV/AIDS being treated with HAART, as well as for the context of Rwanda. Changes included adding risk factors, fear of worsening of the disease, and a lack of counselling on physical activity. Household physical activities were also incorporated. Activities that are not commonly practised by the Rwandan population were removed (e.g. horse-riding, fishing, hunting and animal rearing). Activities were categorised according to their intensity: vigorous, moderate, light, and sitting (Prochaska, Sallis, Sarkin & Calfas 2000).

The modified questionnaire consisted of 16 questions grouped into five sections, which assessed the different categories of physical activities, including leisure-time activities; household activities; walking to and from work; shopping, school and church; as well as occupation-related physical activities. The participants’ personal evaluations of physical activity levels were determined in relation to the World Health Organization (WHO) recommendations. Frequency and duration of participation in these physical activities, as well as factors preventing participants from practising them, were also included.

### Reliability and validity

The original English questionnaires were translated into Kinyarwanda, the language most commonly used by the participants. The questionnaire had been pre-tested for face validity among 15 people living with HV/AIDS and being treated with HAART, who were not included in the main study. The aim was to test how well respondents understood the questions, and how long it took to complete the questionnaire. Content validity of the revised questionnaire was done by experts in the area of physical activity, as well as experts dealing with HIV patients, to ensure that the categories of items that had been added to the questionnaire were relevant.

### Procedure

After obtaining permission from the institutional Ethics Board and the National Ethics Committee of Rwanda, permission was also obtained from the heads of selected health centres in Kigali, Rwanda. Data were collected by one female and one male research assistant who could speak and write both English and Kinyarwanda fluently. Having a female in the research team was very helpful to participants who were not comfortable with a male. Once participants indicated their willingness to take part, the researcher obtained informed written consent from them, after explaining and ensuring that they understood the process. The issues of respect, confidentiality, anonymity and the right to withdraw were explained to the participants.

Data were then gathered before the patients received their HAART treatment. Anthropometric measurements were taken on the same day by the same researcher to ensure intra-rater reliability and measurements were recorded twice and the average taken. The referring of participants with risks of cardiovascular and diabetes was carried out. Advice on the importance of physical activity was given to all participants.

### Data analysis

Completed data were captured on a spread sheet using the Microsoft Excel programme in preparation for analysis. The data were then transferred into the Statistical Package for the Social Sciences (SPSS) Version 19. Descriptive statistics were used to summarise the demographic data, medical profiles and anthropometric profiles, followed by physical activity levels, as well as the factors associated with physical inactivity. The categorical data were given in the form of percentages and frequencies. Means and standard deviations were used for continuous data. Inferential statistics such as the chi-square test were employed in order to test the association between variables, such as level of participation in physical activity and anthropometric profiles, and then variables such as age and gender were associated with physical inactivity (*p* < .05).

## Results

This study investigated a sample of 407 people living with HIV/AIDS and currently on HAART, who attended the three selected health centres located in Kigali city, Rwanda. The majority of people surveyed were females (77%). Participants’ ages ranged from 18 to 76 years, with the mean age being 8.9 (38.82) years. In addition, 54% of the participants were married, and 65% declared that they had only completed primary school. Most (51%) of the participants were self-employed. Of the participants, 37% had spent 6 years and more on HAART. The majority (72%) had CD4 cell counts equal to or more than 350 cells per mm^2^. Approximately 43% of the participants reported having opportunistic diseases, and approximately 63% had neuropathic diseases.

## Anthropometric profiles

According to the anthropometric measurements, the BMI revealed that approximately 40% were in the obese (*n* = 65) and overweight (*n* = 96) categories. The waist circumference revealed that 31% were in the high-risk category of abdominal obesity, and according to the WHR, 43% were in the high-risk category of central obesity. The BMI was found to be significantly associated with age, gender, education and CD4 cell counts (*p* < .05). In addition, the chi-square test showed that there was a statistically significant association between age, gender, education level, occupation, duration on HAART, and waist circumference. A significant association (*p* < .05) was found between gender, occupation, duration on HAART, and WHR profile (Table 1).

**Table 1.** Anthropometric measurements,

	BMI				Waist circumference			WHR		
Variable	Underweight (%)	Normal (%)	Overweight (%)	Obesity (%)	Low risk (%)	Moderate risk (%)	High risk (%)	Low risk (%)	Moderate risk (%)	High risk (%)
Age*										
15–34 years	3	63	22	12	65	10	25	45	22	36
35–54 years	4	54	26	16	53	15	32	38	17	45
55 years and above	0	41	6	53	24	18	59	29	18	53
	*p* = .002*				*p* = .009*			*p* = .430		
Gender*										
Female	4	51	25	21	46	14.4	39.	25.3	19.9	54.8
Male	1.1	77	21	1.1	86	10.5	3.2	86.3	10.5	3.2
	*p* = .000*				*p* = .000*			*p* = .000		
Duration on HAART*										
Only 1 year	3	75	11	11	75	14	11	58	6	36
2–3 years	1	66	17	16	59	26	14	44	25	31
4–5 years	2	53	26	18	56	9	35	35	14	50
6 years and above	21	50	29	16	50	13	33	36	19	44
	*p* = .057				*p* = .042*			*p* = .011*		
CD4 cell counts*										
<200	22	52	19	7	69	10	21	46	14	39
200 and <350	2	61	24	13	62	14	24	48	15	37
≥500	2	55	24	18	52	14	34	26.4	18	37
	*p* = .000*				*p* = .253			*p* = .409	

* Significant at 5% level.

## Levels of physical activity of the study sample

The levels of participation in physical activity were categorised depending on the recommendations for health by WHO. They recommend a total of at least 30 min of moderate-intensity physical activity per day, five or more days a week. It can also be three or more times per week for at least 20 min of vigorous activity in order to prevent the diseases caused by sedentary lifestyles (Miles 2007). The participants were classified as physically active or sedentary, based on the above-mentioned recommendations.

Based on the results, a large percentage of participants were found to be inactive. The highest number (82.6%) of inactive participants was classified as leisure-time physical activity (Table 2). For the age group 15–34 years, 74% were classified as inactive in leisure-time activities, 72% in household activities, 68% in walking, and 61% in work activities. A statistically significant association was found between waist-to-hip ratio and leisure-time physical activity (*p* < .05) as well as gender and leisure-time physical activity (*p* < .05). Although there was no significant association between physical activity levels and exposure to HAART, it was reported that participants on HAART for more than 4 years were inactive (83%). In addition, participants with CD4 cell counts of more than 350 were found to be physically inactive (71%).

**Table 2.** Physical activity levels according to activities (*N* = 407).

Types of activities	Inactive	Active
Leisure time physical activity	336 (83%)	71 (17%)
Household physical activity	289(71%)	118(29%)
Work physical activity	251 (62%)	156 (38%)
Walking to and from work	306 (75%)	101 (25%)

Barriers to participation in vigorous or moderate leisure-time physical activity were assessed for the study sample. The main barriers highlighted by participants were lack of motivation (30.5%) followed by a lack of time (25.3%) and also the fear of worsening the disease (24.3%).

## Discussion

According to Amorosa *et al.* (2005), physical activity has been identified as a method to manage the adverse effects of HAART in people living with HIV/AIDS. Various researchers have indicated that physical activity is likely to be safe for people living with HIV/AIDS (Bopp *et al.* 2003; Roubenoff & Wilson 2001) and participating in physical activity can result in various benefits. Encouraging physical activity is in fact essential for people with HIV/AIDS in order to combat the onset of secondary disorders such as non-communicable diseases. However, the promotion of participation is dependent on the person's HIV/AIDS status, medications, functional capacity and symptoms, and an understanding of the current levels of the group is essential.

In the current study, a majority of the participants (74%) who were inactive were in the 15–34-year age group. This is cause for concern, as literature indicates that physical activity levels tend to decrease as age increases (Sagatun, Kolle, Sigmund, Thoresen & Sogaard 2008). In addition, these participants are considered to be in the productive group in society, and pose a public health challenge if they are unable to contribute to the economy. Despite evidence that participation in physical activity does not negatively affect immune measures, and can have a positive impact on many of the associated conditions of HIV/AIDS (Terry *et al.* 2006), participants do not engage sufficiently in physical activity. In the current study, it was found that the majority of the participants had a CD4 count of 350 and above, indicating the potential to participate in physical activity (Florindo *et al.* 2007). However, more than 70% of the participants were physically inactive.

An added challenge found in this study was that in addition to the high prevalence of physical inactivity, participants had high levels of risk for obesity, thus increasing the opportunity for chronic diseases of lifestyle. The BMI was found to be significantly associated with age, gender, education and CD4 cell counts of the participants (*p* < .05). These risk factors associated with this high level of inactivity are cause for concern among health professionals. In the National Strategic Plan for Rwanda, the Minister of Health has indicated that although a large proportion of patients affected by HIV are receiving treatment, the social, economic and health burdens remain a challenge (HSPR 2009). If this is coupled with an increase in risk factors for non-communicable diseases among the people living with HIV/AIDS, this may result in a double burden of disease for the government.

## Conclusion

There is clear evidence that there is a need for health promotion strategies that target the 15–49-year-old people living with HIV/AIDS. People living with HIV/AIDS are already burdened with additional health costs, and if coupled with associated diseases such as non-communicable disease, the burden could have a disastrous impact. It is, therefore, vital to encourage intervention strategies that focus on promotion of physical activity among people living with HIV/AIDS. It is essential that these intervention strategies have a holistic approach that addresses these people's physical, emotional and psychological well-being.
